# Evaluation of Klebsiella pneumoniae pathogenicity through holistic gene content analysis

**DOI:** 10.1099/mgen.0.001295

**Published:** 2024-09-19

**Authors:** Miyu Isogai, Kumiko Kawamura, Tetsuya Yagi, Shizuo Kayama, Motoyuki Sugai, Yohei Doi, Masahiro Suzuki

**Affiliations:** 1 Department of Integrated Health Sciences, Nagoya University Graduate School of Medicine, Aichi, Japan; 2 Department of Infectious Diseases, Nagoya University Graduate School of Medicine, Nagoya, Japan; 3 Antimicrobial Resistance Research Center, National Institute of Infectious Diseases, Tokyo, Japan; 4 Department of Antimicrobial Resistance, Hiroshima University Graduate School of Biomedical and Health Sciences, Hiroshima, Japan; 5 Department of Microbiology, Fujita Health University School of Medicine, Aichi, Japan; 6 Center for Infectious Disease Research, Fujita Health University, Toyoake, Aichi, Japan; 7 Department of Infectious Diseases, Fujita Health University School of Medicine, Aichi, Japan; 8 Center for Innovative Antimicrobial Therapy, Division of Infectious Diseases, University of Pittsburgh School of Medicine, Pittsburgh, Pennsylvania, USA

**Keywords:** *Galleria mellonella*, *Klebsiella pneumoniae*, genomes, virulence

## Abstract

*Klebsiella pneumoniae* is a Gram-negative bacterium that causes both community- and healthcare-associated infections. Although various virulence factors and highly pathogenic phenotypes have been reported, the pathogenicity of *K. pneumoniae* is still not fully understood. In this study, we utilized whole-genome sequencing data of 168 clinical *K. pneumoniae* strains to assess pathogenicity. This work was based on the concept that the genetic composition of individual genomes (referred to as holistic gene content) of the strains may contribute to their pathogenicity. Holistic gene content analysis revealed two distinct groups of *K. pneumoniae* strains (‘major group’ and ‘minor group’). The minor group included strains with known highly pathogenic clones (ST23, ST375, ST65 and ST86). The minor group had higher rates of capsular genotype K1 and presence of nine specific virulence genes (*rmpA*, *iucA*, *iutA*, *irp2*, *fyuA*, *ybtS*, *iroN*, *allS* and *clbA*) compared to the major group. Pathogenicity was assessed using *Galleria mellonella* larvae. Infection experiments revealed lower survival rates of larvae infected with strains from the minor group, indicating higher virulence. In addition, the minor group had a higher string test positivity rate than the major group. Holistic gene content analysis predicted possession of virulence genes, string test positivity and pathogenicity as observed in the *G. mellonella* infection model. Moreover, the findings suggested the presence of as yet unrecognized genomic elements that are either involved in the acquisition of virulence genes or associated with pathogenicity.

**Impact Statement**

*Klebsiella pneumoniae* strains were classified into two groups based on holistic gene content analysis. The minor group was identified as more virulent through *Galleria mellonella* assays and included strains with previously identified highly pathogenic clones. This group was distinguished not only by the presence of specific virulence genes but also by holistic gene content, suggesting that a complex interplay of various genes contributes to the pathogenicity of *K. pneumoniae*. The insights derived from this analysis underscore the necessity for future investigations that examine the gene contents in their entirety, identify additional virulence genes and elucidate their functional roles in the pathogenesis of *K. pneumoniae* infections.

## Data Summary

The sequencing reads of the clinical strains can be accessed through the BioProject accession numbers PRJDB12075, PRJDB12116, PRJDB14656 and PRJDB17849. The code is available at: https://github.com/suzukimasahiro/HolisticGeneContentAnalysis/.

## Introduction

*Klebsiella pneumoniae* is a Gram-negative rod-shaped bacterium belonging to the order *Enterobacterales*. It is a common opportunistic pathogen that causes nosocomial infections, including urinary tract infection, pneumonia and bloodstream infection [1,2]. In addition, certain *K. pneumoniae* strains were reported to cause community-associated liver abscess along with endophthalmitis in Taiwan in the 1980s, and this group of strains was designated as hypervirulent *K. pneumoniae* (hvKP) [3]. hvKP has since become increasingly prevalent in the Asian–Pacific region and has garnered attention due to its ability to cause severe and invasive infections [4]. Multiple virulence factors and phenotypes of hvKP have been characterized. For example, strains carrying the *rmpA* gene (involved in the regulation of capsular production) are related to hypermucoviscosity [5], strains carrying the *iucABCDiutA* loci (involved in iron uptake) are related to liver abscess, endophthalmitis and other metastatic infections [6], and strains with capsular genotypes K1 and K2 are associated with resistance of phagocytosis [7]. Not all strains with these virulence genes exhibit high pathogenicity, and conversely, some strains lacking these genes are highly pathogenic. Thus, the pathogenicity of *K. pneumoniae* is still not fully understood [8].

The genetic composition of individual genomes (referred to as holistic gene content in this study) of *K. pneumoniae* strains, alongside the aforementioned specific virulence genes, holds promise for potential use in estimating virulence, facilitated by improving access to whole-genome sequencing (WGS) [9]. WGS, with advances in the technologies and lowering of costs, is increasingly used in *K. pneumoniae* studies. *In silico* analyses utilizing WGS data encompass sequence type (ST) identification through multilocus sequence typing (MLST), capsular genotype determination using the *wzi* analysis, and identification of virulence and resistance genes [6,10,13]. Additionally, phylogenetic analysis based on core genome SNPs (cgSNPs) using WGS data is widely employed for epidemiological investigations and detection of transmission routes [14]. However, current analyses often utilize limited regions of the *K. pneumoniae* genome, such as those with registered reference sequences or core genomic regions, and do not fully leverage WGS data that are available.

To address this limitation, innovative approaches in processing WGS data are needed. The pan-genome approach is a method that simplifies the handling of WGS data [15]. In this approach, WGS data can be encoded into binary sequences determined by the presence or absence of ORFs. In addition, the outcomes of the analysis, which are binary sequences with tens of thousands of ORFs, can be visualized and represented on a simple two-dimensional plane using principal component analysis (PCA). PCA is a data extraction and analysis method widely employed in the field of biological research [16]. Use of unsupervised machine learning techniques like k-means clustering represents an alternative method for processing WGS data. In this approach, binary sequences generated from WGS data are grouped into clusters using the k-means algorithm. Compared with standard phylogenetic analysis, clustering analysis offers a more accessible means of understanding population structures. Notably, phylogenetic analysis necessitates the establishment of a cutoff value, a potentially arbitrary task, whereas clustering analysis operates without such a requirement. This not only streamlines the analytical process but also enhances the interpretability of population dynamics derived from WGS data.

However, predicting pathogenicity of *K. pneumoniae* solely from WGS remains challenging. Therefore, pathogenicity assessment employs various methods, such as the string test, biofilm production assay, serum resistance assay, and *Galleria mellonella* and murine infection models. Among them, *G. mellonella* larvae have garnered considerable attention within the scientific community for use in infection models due to the substantial similarities between the innate immune system of *G. mellonella* larvae and that of mammals. Moreover, utilizing *G. mellonella* larvae is a cost-effective alternative to traditional animal models based on financial, technical and ethical considerations [17]. It has been reported that murine models, as opposed to *G. mellonella* models, are more suitable for accurately evaluating pathogenicity of *K. pneumoniae* [18]. Nonetheless, combining *G. mellonella* infection experiments with the string test has been demonstrated to significantly improve the identification of pathogenicity [19].

In this study, we aimed to provide novel insights into the relationship between genomes and pathogenicity of *K. pneumoniae*. To achieve this, clustering analysis was utilized to categorize *K. pneumoniae* strains according to their holistic gene content. Subsequently, these strains underwent screening for pathogenicity using the string test and *G. mellonella* infection experiment.

## Methods

### Bacterial strains

A total of 168 non-redundant clinical strains of *K. pneumoniae* were collected from January 2015 to April 2018 by MIROKU Medical Laboratory, Hiroshima University and Nagoya University Hospital, all located in Japan. Among them, 85 strains were isolated from blood, 37 from respiratory tract (29 from sputum, five from nasopharyngeal swab, two from tracheal aspirate, one from bronchoalveolar lavage), 24 from urine (12 from urinary catheter, ten from midstream urine and two from non-classified urine samples), two from pus, one from skin and one from bile. The sources of 18 strains were unidentified or missing (Tables 1 and S1, available in the online version of this article). Further details of the strains were not available. Identification of these strains as *K. pneumoniae* was performed using OrthoANI version 0.5.0 (https://github.com/althonos/orthoani) based on the WGS data [20]. The cutoff value for the average nucleotide identity (ANI) used for bacterial species identification was set at 95%, with * K. pneumoniae* HS11286 (accession number: CP003200) as the reference genome.

**Table 1.** Sequence type (ST) and source of strains in each group

	Major group						Minor group					
ST	Blood culture	Respiratory tract specimens	Urinary tract-related specimens	Others	Missing	Subtotal	Blood culture	Respiratory tract specimens	Urinary tract-related specimens	Others	Missing	Subtotal
11			1		3	4						
12	1		1			2						
13		1	1			2						
17			3			3						
20	2	1				3						
23							2	3	1	1	2	9
25	5		1			6						
29	4	2	1			7	1	1				2
34	2	1				3						
36	3		1			4						
37	5	3			3	11						
45	1				1	2						
65								2				2
147					2	2						
247	2	1				3						
268							2	3	1			6
280	1	1	2			4						
307			1		1	2						
375							3					3
412	1					1		4	3			7
485	1	1				2						
528	2					2						
730	1			1		2						
893							1				1	2
1569		1	1			2						
3430	2					2						
5576					2	2						
Others	42	10	6	2	2	62	1	2			1	4
Total	75	22	19	3	14	133	10	15	5	1	4	35

### WGS and assembly

Illumina short-read sequencing was conducted on the 168 strains. Genomic DNA extraction was performed using the QIAamp DNA Mini Kit (Qiagen) or the Gentra Puregene Yeast/Bact.Kit (Qiagen), followed by quantification of DNA concentrations using the Quantus Fluorometer (Promega). Sequencing libraries were prepared using either the Nextera XT DNA Sample Preparation kit (Illumina) or the QIAseq FX DNA Library kit (Qiagen), following the manufacturers’ instructions. Subsequently, the libraries were sequenced with the Illumina MiSeq or NextSeq 2000 sequencer using MiSeq Reagent Kit v3 (600 cycle) [21] or NextSeq 1000/2000 P2 Reagents (300 cycle) (Table S1) [22].

To enhance data quality, low-quality raw reads were trimmed using fastp version 0.23.1 (https://github.com/OpenGene/fastp) and assembled either through the A5-miseq pipeline version 20 160 825 (https://sourceforge.net/p/ngopt/wiki/A5PipelineREADME/) or SPAdes version 3.13.1 (https://github.com/ablab/spades) .

The sequencing reads of the clinical strains can be accessed through the BioProject accession numbers PRJDB12075, PRJDB12116, PRJDB14656 and PRJDB17849. Among them, 14 strains in PRJDB12075 were collected and sequenced by Hiroshima University and four strains in PRJDB12116 were collected by Hiroshima University and sequenced by Fujita Health University. All other isolates were sequenced at Fujita Health University (Table S1).

### Detection of ORFs and binary sequence generation

Identification of ORFs and the construction of binary sequences based on gene presence/absence were performed using a custom Python script (https://github.com/suzukimasahiro/OSNAp) [23]. Briefly, the sequencing reads of clinical strains were annotated through DFAST (https://dfast.ddbj.nig.ac.jp/dfc/distribution/) and dissected into ORFs. A hypothetical sequencing read encompassing all elemental ORFs was constructed as the reference. The presence or absence of ORFs in the sequencing reads was determined by BLASTn, based on the reference sequence. The results of BLASTn were converted to 1 if the ORF was present and 0 if absent, then described as a binary sequence.

### PCA

Data analysis was performed by an in-house python program (https://github.com/suzukimasahiro/HolisticGeneContentAnalysis/). For feature selection, the binary sequence was reconstructed for ORFs that were possessed by 10–90% of clinical strains used in this study and reduced to two dimensions using PCA from scikit-learn library version 1.0.2 (https://github.com/scikit-learn/scikit-learn). The strains were plotted on the PCA plot based on the first and second principal components using matplotlib library version 1.23.2 (https://github.com/matplotlib/matplotlib).

### Identification of genotypes and virulence genes

STs were determined using the locally installed MLST 2.0 (version 2.0.9) software obtained from the Center for Genomic Epidemiology server (https://cge.food.dtu.dk/services/MLST/) [12]. Capsular genotypes (exact matches) and virulence genes (with 90% identity and 60% minimum length) were determined using BLASTn based on the online database of the Pasteur Institute MLST for *K. pneumoniae* (https://bigsdb.pasteur.fr/klebsiella/) [6,10, 13]. To visualize the distribution of ORFs in each strain, we conducted a pan-genome analysis using Roary (http://sanger-pathogens.github.io/Roary/). Additionally, to clearly show the distribution of genotypes among the strains used in this study, a cgSNP analysis was performed. This was done using reference strains introduced by Holt *et al*. (Table S2), with HS11286 as the reference for mapping, following the SNIPPY pipeline (https://github.com/tseemann/snippy) [24]. The phylogenetic tree was visualized using MEGA7 (https://www.megasoftware.net/).

### String test

The string test was conducted to detect the hypermucoviscous phenotype. The strains were cultured overnight at 37 °C, after which the colonies were elongated using a plastic needle. Strains exhibiting strings with a length of 5 mm or more were considered positive. Positivity rates were calculated for each group [25,26].

### Selection of strains for virulence assay

First, unsupervised clustering analysis was conducted using the data of the 168 strains after dimensionality reduction. Silhouette analysis, facilitated by the Silhouette_score from the python 3.8.5 scikit-learn library version 1.2.1, was employed to determine the optimal number of clusters. Subsequently, GaussianMixture from the scikit-learn library was utilized for cluster formation and cluster centre calculation. The k-means algorithm was applied to initialize the parameters of the Gaussian distribution, and the random state was set to ensure reproducibility.

Next, from each formed k-means cluster, two strains that were the closest and second closest from the centre of the corresponding cluster, with distances computed using the NumPy library version 1.23.2, were selected to be tested in *G. mellonella* infection experiments. The selection procedures were performed using an in-house program (https://github.com/suzukimasahiro/HolisticGeneContentAnalysis/).

For visual representation, the strains and cluster centres were plotted using the matplotlib library version 3.4.1 (https://github.com/matplotlib/matplotlib).

### *G. mellonella* larvae

*G. mellonella* larvae were reared on a diet consisting of 80 g 60% sugared water, 80 g glycerol, 200 g powdered mouse diet and 100 µl Altosid (10% Methoprene; Earth) and maintained at 30 °C in an incubator [27]. For the infection experiments, late-instar larvae approximately 2 cm in length were used [28].

### *G. mellonella* infection experiment

*G. mellonella* infection experiments were performed with modifications to previously reported protocols [27,30] using the 36 selected strains. Specifically, bacteria were cultured at 37 °C on trypticase soy agar (Eiken Chemical) until reaching the late exponential phase. Subsequently, each strain was suspended in PBS (Fujifilm Wako Pure Chemical) to prepare a McFarland 0.5 (2×10^8^ c.f.u. ml^−1^) suspension. Serial 10-fold dilutions were prepared, ranging from 2×10^8^ to 2×10^3^ c.f.u. ml^−1^. Ten randomly selected *G. mellonella* larvae were inoculated with 2×10^7^ c.f.u. ml^−1^ (10 µl aliquot) into the haemocoel through the rear left proleg, using a 30-gauge dental needle (Terumo). PBS inoculated larvae were included as a negative control. Infected larvae were then placed in Petri dishes and incubated in the dark at 37 °C without food for 4 days. Larvae were examined every 24 h, and daily mortality assessments were conducted, considering no response to stimulation as an indication of death. The experiments were repeated three times. The inocula were verified by plating 10 µl of each 2×10^3^ and 2×10^4^ c.f.u. ml^−1^ bacterial solution on trypticase soy agar, followed by colony counting. If the inoculum deviated outside the range of 2±1×10^5^ c.f.u. per larva, the experiment was repeated.

Survival curves for each group were generated using the Kaplan–Meier method with R version 4.1.0. For each strain, the average survival rate was determined by calculating the mean of the cumulative survival rates of *G. mellonella* larvae over 4 days in three experiments.

### Statistical analysis

Positivity rates of virulence genes among each group were assessed using Fisher’s exact test, while survival trends were analysed through the Log-rank test. All statistical analyses were conducted using R version 4.1.0, and significance was established with a two-sided threshold of *P*<0.05.

## Results

### PCA plot

**Fig. 1.**
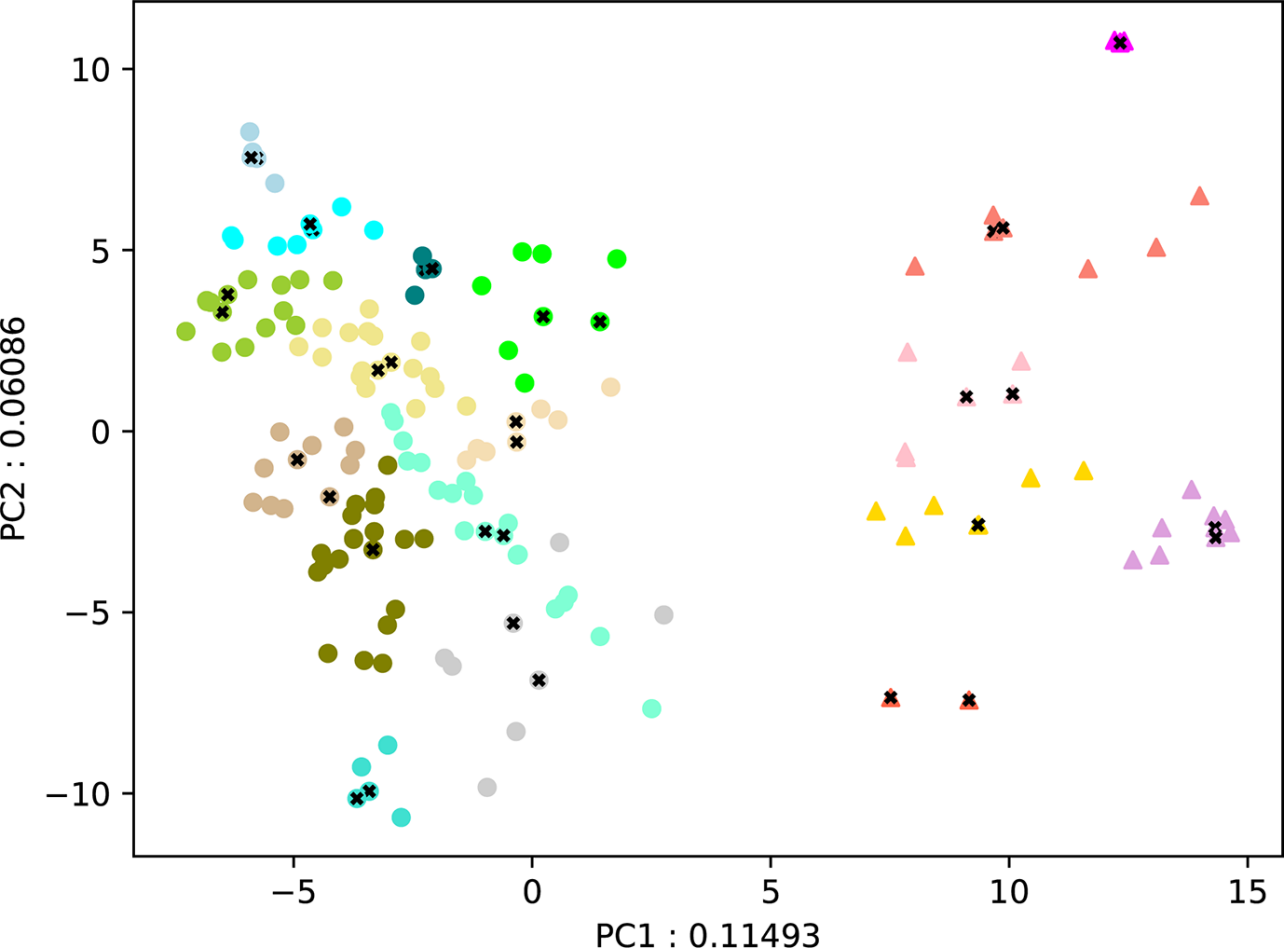
PCA plot of 168 clinical strains. Distribution of genotypes of 168 strains is plotted based on first and second principal components obtained from PCA. The major group, consisting of 133 strains, is denoted by circles, while the minor group, composed of 35 strains, is represented by triangles. Various subgroups, identified for selecting strains for the *G. mellonella* assay, are distinguished by different colours. The optimal number of subgroups (18) was determined through silhouette analysis. Additionally, strains utilized in the *G. mellonella* assay are indicated by crosses. Note that no batch effect correction for the sequencing procedure was performed for this analysis. As a result, there may be effects due to batch variation.

The 168 strains were divided into two groups based on the first principal component (PC1): those with a PC1 value less than 2.8 (major group) and those with a value greater than 7.2 (minor group), as shown in the PCA plot in Fig. 1. Within the major group, the predominant source was blood (75 strains, 56.4%), whereas in the minor group, respiratory tract specimens were the primary source (15 strains, 42.9%), followed by blood (ten strains, 28.6%) (Table 1).

### Genotypes and virulence genes

**Fig. 2.**
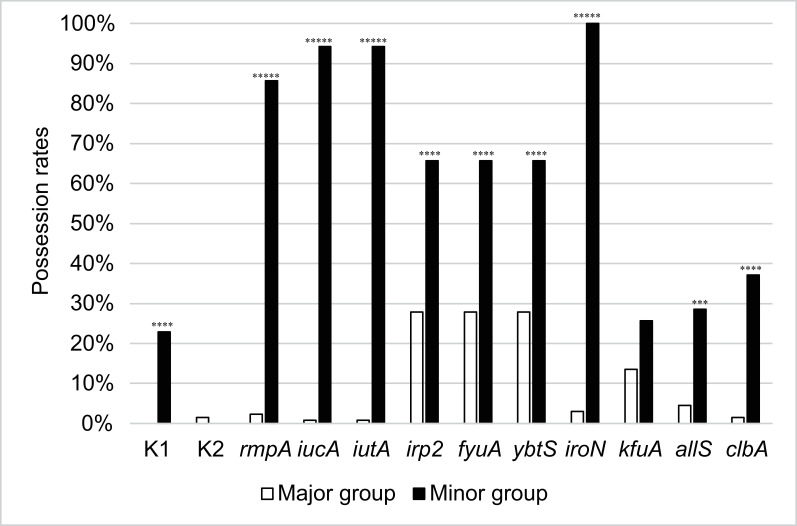
Comparison of virulence gene possession rates between the major and minor groups. The graph displays the rates of capsular genotype K1/K2 strains and the possession rates of ten cardinal virulence genes. Statistical analysis comparing possession rates between the major and minor groups was conducted using Fisher’s exact test. Significance levels are denoted as follows: *P<0.05, ***P<0.001, ****P<0.0001, *****P<1×10^−10^.

The 133 strains in the major group were assigned to 84 STs, while the 35 strains in the minor group were classified into 11 STs (Tables 1 and S3a, S3b). Fig. 2 displays the positivity rates of capsular genotypes K1/K2 and ten cardinal virulence genes in each group. The positivity rate of capsular genotype K1 was 22.9% (8/35) in the minor group, with all K1 strains belonging to the hvKp lineage of ST23. In the major group, there were no cases of genotype K1 (0/133). That of K2 was 1.5% (2/133) in the major group and zero in the minor group (0/35). For other virulence genes (*rmpA*, *iucA*, *iutA*, *irp2*, *fyuA*, *ybtS*, *iroN*, *allS* and *clbA*), the possession rates were significantly higher in the minor group (Table 2).

**Table 2.** Positive rate of virulence genes in the major and minor groups

Virulence gene	Major group (%)	Minor group (%)	*P* value
*rmpA*	2.3	85.7	2.2×10^−16^
*iucA*	0.8	94.3	2.2×10^−16^
*iutA*	0.8	94.3	2.2×10^−16^
*irp2*	27.8	65.7	5.6×10^−5^
*fyuA*	27.8	65.7	5.6×10^−5^
*ybtS*	27.8	65.7	5.6×10^−5^
*iroN*	3.0	100	2.2×10^−16^
*kfuA*	13.5	25.7	0.12
*allS*	4.5	28.6	0.00015
*clbA*	1.5	37.1	1.4×10^−8^

A higher genetic diversity was observed among the strains used in this study on the phylogenetic tree (Fig. S1). On the tree, strains from minor groups were distributed across different clusters for each ST type. On the other hand, the contents of ORFs showed a similar tendency among strains within the minor group from pan genome analysis (Fig. S2).

Because carriage of virulence genes was significantly more common in the minor group for all virulence genes of interest, we considered the possibility that the result of the PCA was significantly influenced by the virulence genes. We therefore repeated PCA on the data set excluding the ORFs corresponding to these virulence genes. As a result, the distribution of strains remained largely unchanged, and the composition of the major and minor groups persisted (Fig. S3).

### *G. mellonella* infection experiment

Silhouette analysis indicated that the optimal number of clusters was 18 (the major group: 12 clusters, the minor group: six clusters) (Fig. 1). Subsequently, two strains in close proximity to the centre of each cluster were identified for each of the 18 clusters, resulting in the selection of 36 strains (the major group: 24 strains, the minor group: 12 strains) (Fig. 1). The *G. mellonella* infection experiment was then conducted using these 36 strains.

**Fig. 3.**
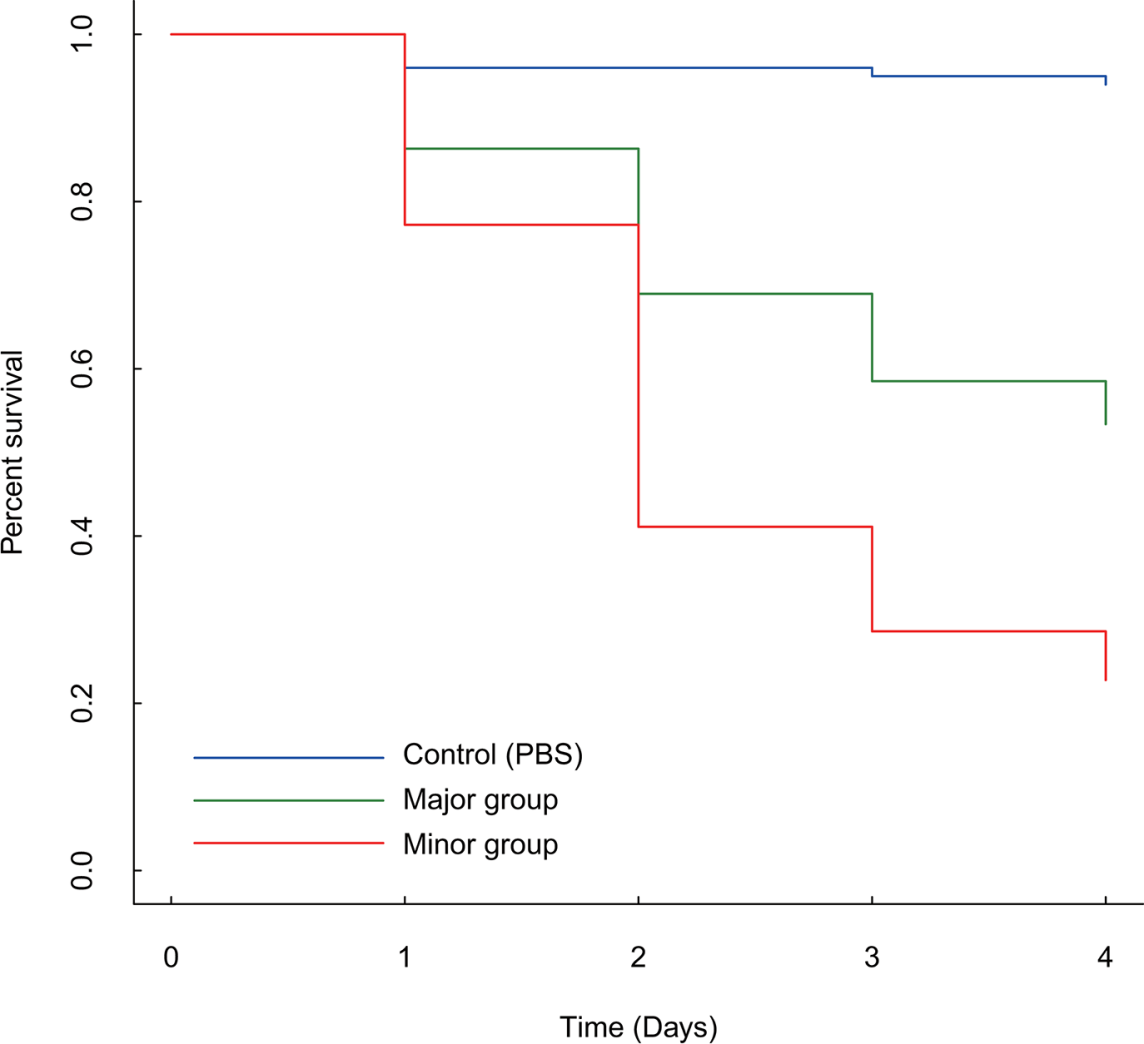
Survival curves of *G. mellonella* larvae for each group. The result of *G. mellonella* infection experiment is shown in survival curves using the Kaplan–Meier method. The survival rate of the minor group (indicated by red line) was lower than that of the major group (indicated by green line) (*P*=2.0×10^−16^, Log-rank test).

The survival curves of *G. mellonella* larvae in each group are depicted in Figs 3 and S4. The survival of *G. mellonella* infected with the strains in the minor group was significantly lower compared with that in the major group (*P*=2.0×10^−16^, Log-rank test).

### String test

Among the 168 strains, the positivity rates of the string test were 13.5% (18/133) in the major group and 51.4% (18/35) in the minor group (*P*=6.9×10^−6^) (Table S3a, S3b). However, there was no correlation between the string test and the *G. mellonella* assay results, with a correlation coefficient of −0.137. Additionally, weak correlations were observed between the string test results and some virulence genes (*rmpA*, *iucA*, *iutA* and *iroN*), with correlation coefficients ranging from 0.351 to 0.366.

## Discussion

*K. pneumoniae* is an important pathogen responsible for both community- and healthcare-associated infections. Common presentations for the former include liver abscess, endophthalmitis and community-acquired pneumonia, whereas healthcare-associated infections often manifest as urinary tract infections, hospital-acquired or ventilator-associated pneumonia, and bloodstream infections. Although various virulence factors and highly pathogenic phenotypes have been reported, the pathogenicity of *K. pneumoniae* is still not fully elucidated. In this study, we implemented holistic gene content analysis to categorize 168 *K*. *pneumoniae* clinical strains and examined whether the resulting groups correlated with pathogenicity.

The *K. pneumoniae* population is known to be highly heterogeneous, consisting of more than 7400 STs. The strains used in this study also showed high heterogeneity based on cgSNP phylogenetic analysis. Among the many STs, the majority of hvKp belong to several specific lineages. In our study involving 168 strains, clustering analysis based on total gene content yielded two distinct groups of *K. pneumoniae* strains: the major group and the minor group. Notably, the minor group comprised strains with ST23 (nine strains), ST375 (three strains), ST65 (two strains) and ST86 (one strain), all of which have been previously identified as hvKp lineages [9,31]. Although strains in the minor cluster did not show relatedness on the cgSNP phylogenetic tree, they exhibited similar patterns in the pan genome analysis. These findings suggest strongly that virulent lineages possess distinct and specific gene content profiles.

Within the minor group, capsular genotype K1 and the nine virulence genes (*rmpA*, *iucA*, *iutA*, *irp2*, *fyuA*, *ybtS*, *iroN*, *allS* and *clbA*) were significantly more common than those in the major group. These factors collectively contribute to virulence, with the capsule providing protection against immunological responses (capsular genotype K1 and *rmpA*) [25,32, 33], iron being essential for bacterial growth and replication (*iucA*, *iutA*, *irp2*, *fyuA*, *ybtS* and *iroN*) [34,37], allantoin-utilizing capability aiding competition for nitrogen sources (*allS*) [3,38] and colibactin inducing DNA damage in host cells (*clbA*) [39,42]. In light of these data, it is reasonable to infer that strains in the minor group exhibit heightened pathogenicity compared to those in the major group. The results from the *G. mellonella* assay also support the notion of increased virulence of the minor group.

Although the minor group also had higher string test positivity, the correlations between string test results and the *G. mellonella* assay and virulence genes were weak or not observed. Hypermucoviscosity might depend on the overall gene content, but this study did not show a relationship between hypermucoviscosity and virulence.

Nonetheless, the classification of strains into major and minor groups does not depend solely on the presence of virulence genes; rather, it is contingent upon the total gene content within their genomes. While PCA inherently sacrifices some interpretability of underlying features, making it challenging to precisely define the nature of each principal component [43], our results strongly underscore the significance of total gene content in this classification. It is plausible that specific genomic contents are either directly involved in the acquisition of virulence genes or are associated with known virulence genes. Further investigation is warranted to elucidate these potential genomic regions and their roles in virulence. Additionally, it has been reported that *G. mellonella* larvae may be susceptible to an unidentified virulence factor in *K. pneumoniae* [19]. In our study, some strains possessing virulence genes were not virulent to *G. mellonella*, while some strains exhibited the opposite behaviour. Considering these findings, it is conceivable that there are undiscovered genomic contents associated with pathogenicity, extending beyond the scope of known virulence genes.

Higher virulence genotypes of *K. pneumoniae* were discerned through holistic gene content analysis. While the minor group representing hvKP strains did exhibit a tendency to harbour known virulence genes, it is noteworthy that this cluster was characterized by numerous genes beyond those recognized as virulence factors. Consequently, it is conceivable that unknown virulence genes may also be present among these additional genes. Exploration of these gene contents holds the potential to unveil unique genetic elements that further characterize higher virulence strains, thereby contributing to a more comprehensive understanding of the pathogenicity of *K. pneumoniae*.

We acknowledge several limitations of the study. First, the clinical strains used in the study had been de-identified irreversibly, and thus we did not have access to the underlying metadata. Second, no batch effect correction for the sequencing procedure was performed for this analysis. As a result, there may be effects due to batch variation.

## Conclusions

This study successfully demonstrated the association of *K. pneumoniae* pathogenicity and their genomes by integrating genomics and experimental virulence assays. Through clustering analysis of WGS data, we identified two distinct groups of *K. pneumoniae,* with the minor group exhibiting a higher prevalence of known virulence genes and more pronounced pathogenicity in * G. mellonella* models. This underscores the importance of gene content in determining bacterial virulence. Our findings highlight the promise of combining holistic gene content analysis with experimental models to fully appreciate the nuances of bacterial pathogenicity. Future research should focus on total gene content and uncovering these additional genetic elements and exploring their roles in *K. pneumoniae* infections, potentially leading to more effective strategies for managing and treating these infections.

## supplementary material

10.1099/mgen.0.001295 Uncited Fig. S1.

10.1099/mgen.0.001295 Uncited Table S1.

## Abbreviations

cgSNP: core genome SNP
hvKP: hypervirulent *K. pneumoniae*
MLST: multilocus sequence typing
PCA: principal component analysis
ST: sequence type
WGS: whole-genome sequencing
