# Supplementary material for: Evaluation of Klebsiella pneumoniae pathogenicity through holistic gene content analysis
Source: Microb Genom. 2024 Sep 19;10(9):001295. doi: 10.1099/mgen.0.001295 (PMC11571079; doi:10.1099/mgen.0.001295)
Supplement: Uncited Fig. S1. [file mgen-10-01295-s001.pdf]

Figure S1

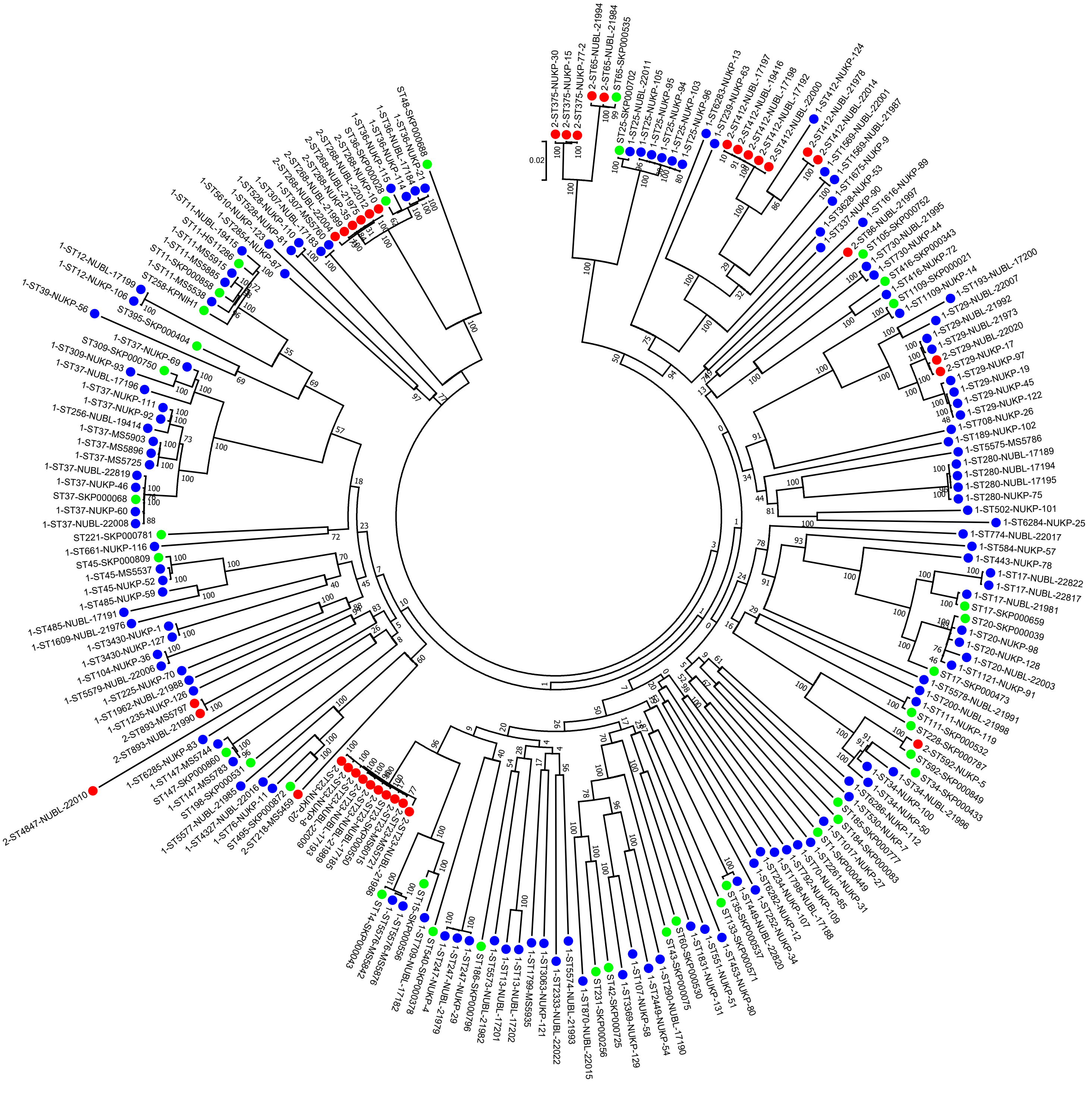

Figure S1. Phylogenetic tree based on cgSNP analysis.

Strains in the Major group are marked in blue, those in the Minor group are marked in red, and the reference strains from Holt et al. are marked in green.

Figure S2

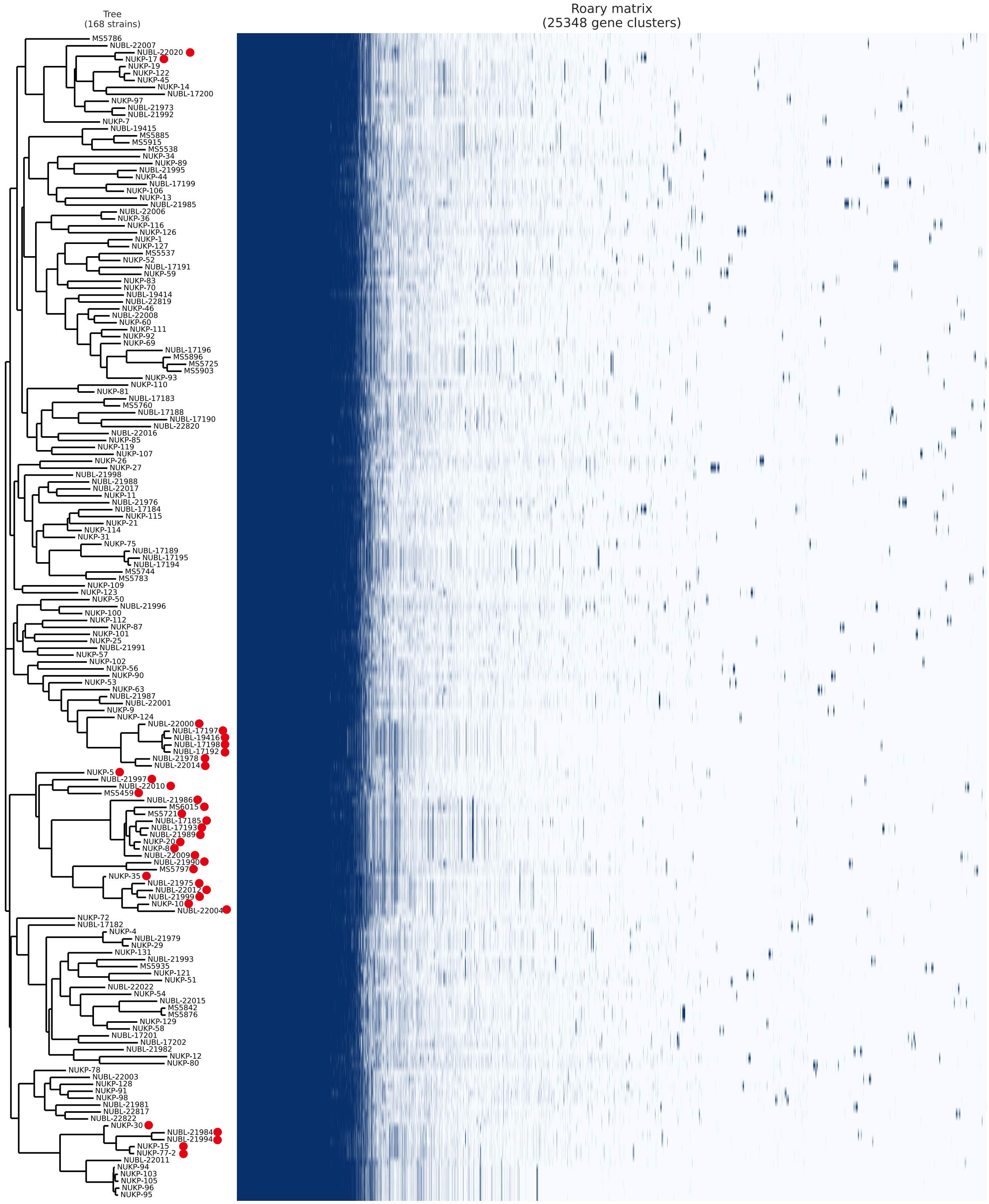

Figure S2. Gene contents based on pan genome analysis.

Strains in the Minor group are marked in red. The dendrogram was generated automatically by Roary based on the results of the pan-genome analysis.

Figure S3

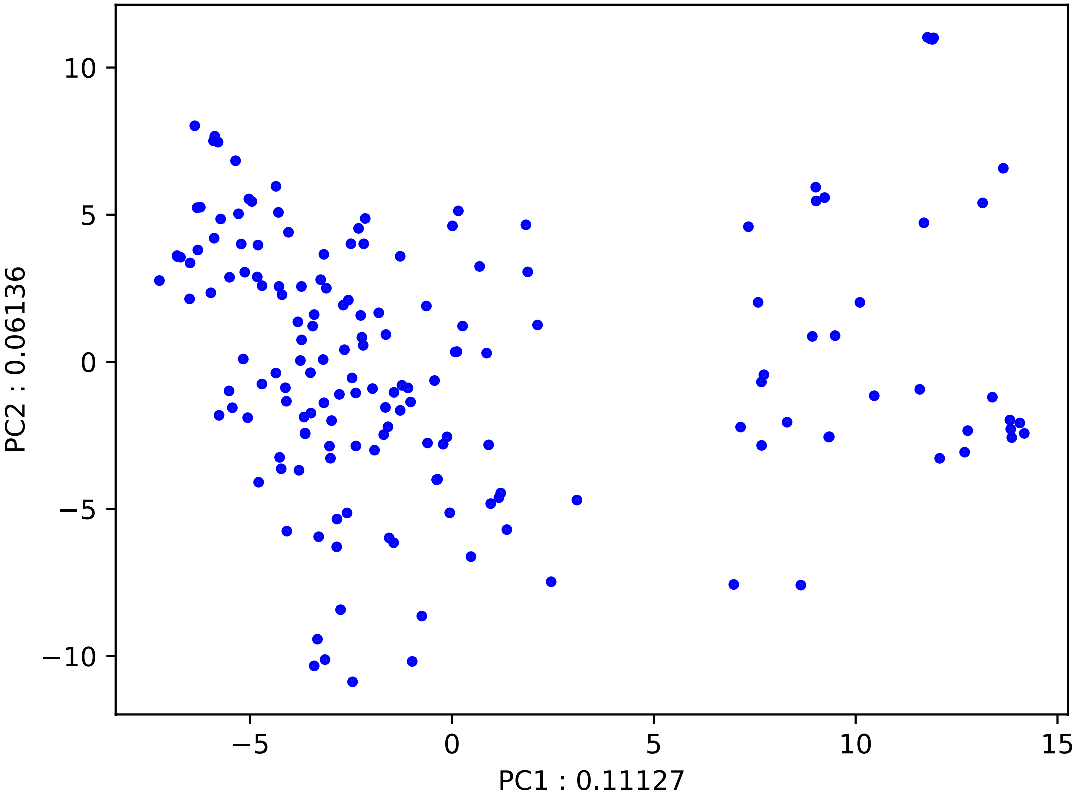

Figure S3. PCA plot of 168 clinical strains without virulence genes.

Distribution of genotypes of 168 strains are plotted based on 1st and 2nd principal component obtained from PCA without virulence genes (capsular genotype K1, *rmpA*, *iucA*, *iutA*, *irp2*, *fyuA*, *ybtS*, *iroN*, *allS*, and *clbA*). The distribution of strains remained almost unchanged, and the composition of the major and minor groups persisted.

Figure S4A

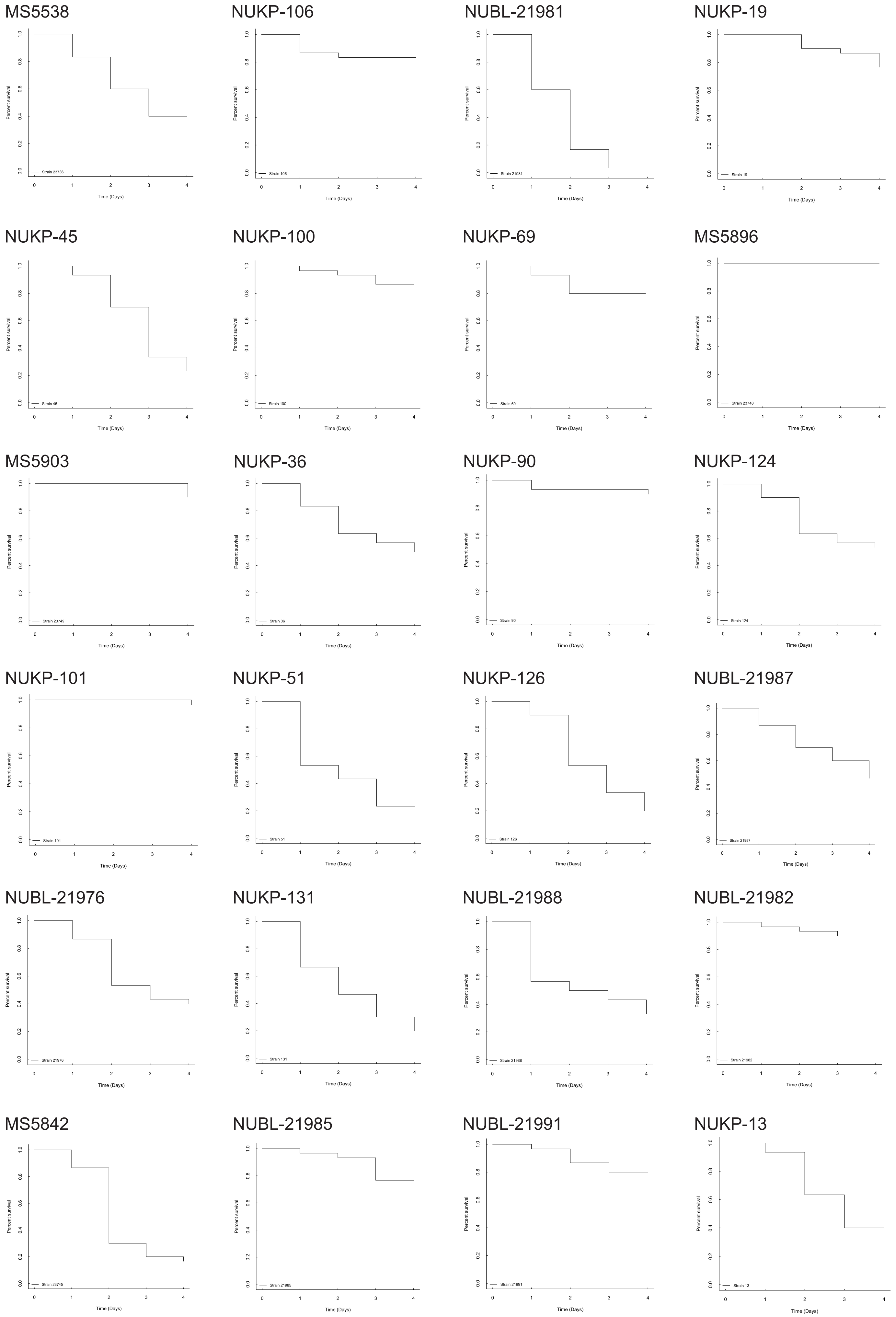
MS5538
NUKP-106
NUBL-21981
NUKP-19
NUKP-45
NUKP-100
NUKP-69
MS5896
MS5903
NUKP-36
NUKP-90
NUKP-124
NUKP-101
NUKP-51
NUKP-126
NUBL-21987
NUBL-21976
NUKP-131
NUBL-21988
NUBL-21982
MS5842
NUBL-21985
NUBL-21991
NUKP-13
Percent survival
Time (Days)

Figure S4B

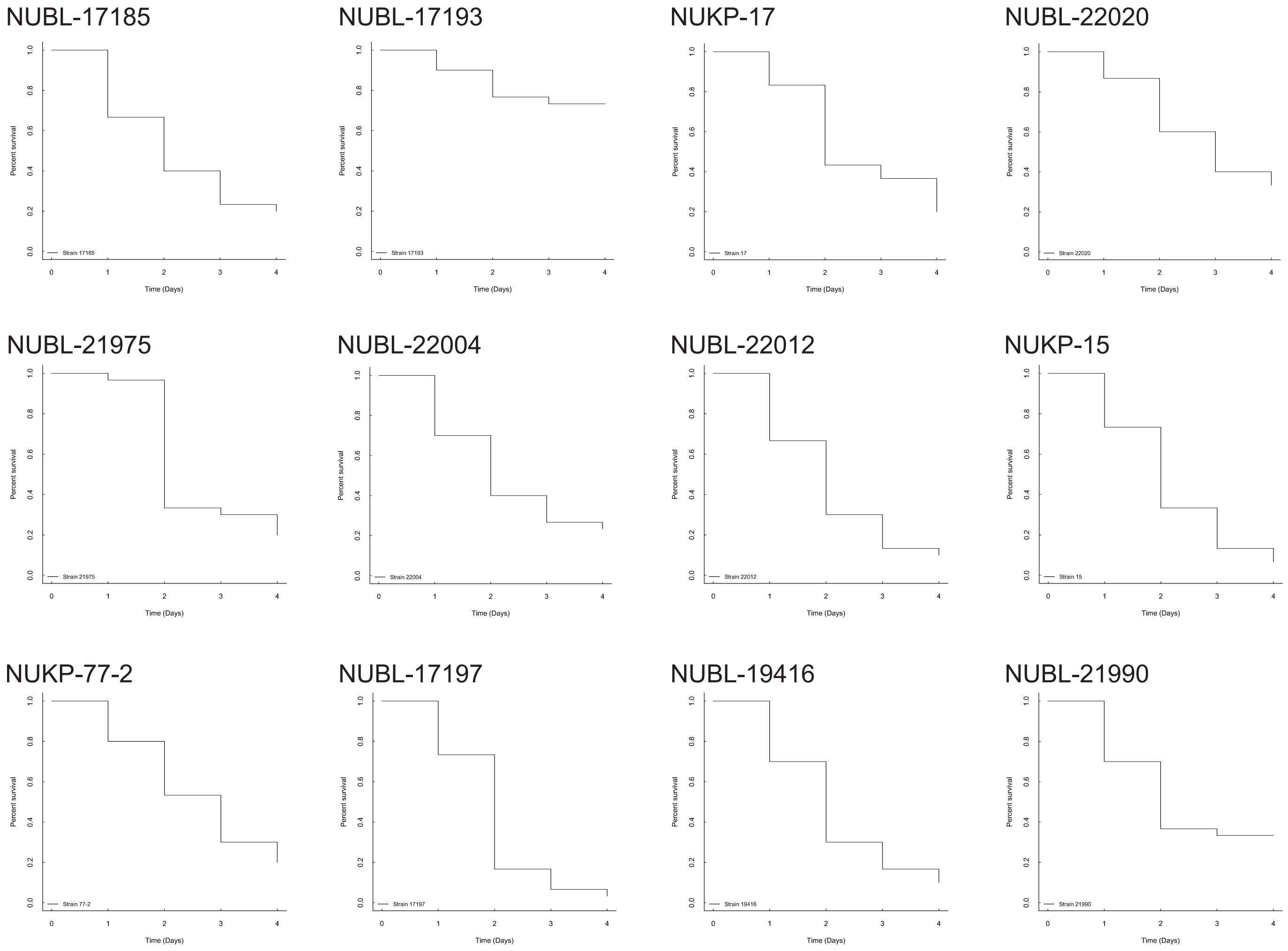

Figure S4. Survival curve for each strain.

The result of *G. mellonella* infection experiment for each strain is shown in survival curves using the Kaplan-Meier method. A: Major group. B: Minor group.
